# Monitoring Contractile Cardiomyocytes via Impedance Using Multipurpose Thin Film Ruthenium Oxide Electrodes

**DOI:** 10.3390/s21041433

**Published:** 2021-02-18

**Authors:** Esther Tanumihardja, Douwe S. de Bruijn, Rolf H. Slaats, Wouter Olthuis, Albert van den Berg

**Affiliations:** 1 BIOS Lab on a Chip Group, Max Planck Centre for Complex Fluid Dynamics and Technical Medical Centre, MESA+ Institute for Nanotechnology, University of Twente, 7500 AE Enschede, The Netherlands; d.s.debruijn@utwente.nl (D.S.d.B.); w.olthuis@utwente.nl (W.O.); a.vandenberg@utwente.nl (A.v.d.B.); 2 Applied Stem Cell Technologies Group, Technical Medical Centre, University of Twente, 7500 AE Enschede, The Netherlands; r.h.slaats@utwente.nl

**Keywords:** ruthenium oxide, electrical impedance spectroscopy, cardiomyocytes, Fourier analysis

## Abstract

A ruthenium oxide (RuOx) electrode was used to monitor contractile events of human pluripotent stem cells-derived cardiomyocytes (hPSC-CMs) through electrical impedance spectroscopy (EIS). Using RuOx electrodes presents an advantage over standard thin film Pt electrodes because the RuOx electrodes can also be used as electrochemical sensor for pH, O_2_, and nitric oxide, providing multisensory functionality with the same electrode. First, the EIS signal was validated in an optically transparent well-plate setup using Pt wire electrodes. This way, visual data could be recorded simultaneously. Frequency analyses of both EIS and the visual data revealed almost identical frequency components. This suggests both the EIS and visual data captured the similar events of the beating of (an area of) hPSC-CMs. Similar EIS measurement was then performed using the RuOx electrode, which yielded comparable signal and periodicity. This mode of operation adds to the versatility of the RuOx electrode’s use in in vitro studies.

## 1. Introduction

In vitro cell cultures are a growing class of models at the center of biomedical research. Many devices have been developed to faithfully replicate human tissues, organs, or organ systems [1,2,3]. More and more of these devices also include sensors to obtain readouts from the living cells [4,5,6]. Such integrated sensors can offer valuable, real-time information without terminating or interrupting the experiments to obtain samples. A popular sensor for such purpose is a metal oxide-based electrochemical sensor. For instance, many works have shown different electrochemical modes of ruthenium oxide (RuOx) electrodes and their applications in in vitro studies. The electrode has been reported as a potentiometric pH electrode [7,8], an amperometric oxygen electrode [9], an amperometric nitric oxide electrode [10,11], as well as an electrophyiosology electrode [12]. This work presents another mode of operation, using the thin film RuOx electrode to observe the contractile activities of a human pluripotent stem cell-derived cardiomyocytes (hPSC-CMs) monolayer through electrical impedance spectroscopy (EIS), demonstrating the versatility of the presented RuOx electrode.

Cardiomyocytes’ contractile performance is surely one of the most important parameters describing the cardiac function. However, the performance of in vitro cardiomyocytes cultures cannot be described using (all of) the typical parameters used for their in vivo counterparts (e.g., certain volumes, pressures, etc.), due to the different dimensionality and absence of confounding factors (e.g., neurohormonal activity) [13]. Therefore, in vitro studies of cardiomyocytes mostly rely on (cellular) electrophysiology, contraction force, and mechanical contractile movements/patterns [13,14,15] to assess the cells’ function. The mechanical contractile movements are among the most straightforward parameters that can depict cardiac function and health [16,17]. The contraction’s periodicity, speed, and direction have been used to describe the function of stem cells-derived cardiomyocytes, their maturity, and cardiotoxicity of certain drugs [14,17,18]. The simplicity of the parameter and the many possibilities for its readout make it an attractive parameter to add to many in vitro studies.

We came to this realization during our cardiomyocytes metabolism study, where the RuOx electrode was used to monitor the cells’ acidification rates and oxygen consumption rates [9]. Since the measured metabolic rates are expected to correlate to the cells’ function or activity, the cells’ contraction rate can serve as a strategic parameter to gain additional insight into the measured metabolic rates. Consequently, we further diversified the functionalities of the used RuOx electrode to also monitor the cells’ mechanical behavior.

A versatile electrode is undoubtedly beneficial in any setting. Fabricating and operating one electrode (type) generally reduces footprint in material, time, electrode cleaning/conditioning steps, and, consequently, cost. The same benefits also apply to the possibility to use one setup for many different applications. On top of that, there are several specific advantages that a multipurpose electrode can offer for in vitro studies of biological cells. An improved spatial resolution can enable the probing of multiple analytes from one specific cell/cell cluster [9,19]. Also, when working on the lower end of in vitro devices scale [20], it is conceivable that there are instances where a device can only include one (set of) electrode(s). In such a case, the option to extract multiple parameters from the same cell(s) would be using a multipurpose electrode.

In time-sensitive experiments, it might be preferred to obtain simultaneous readings of the different parameters. Unfortunately, in most cases, it is impossible to operate different modes using the same electrode simultaneously. However, the different modes of operation can often be performed using one piece of equipment which allows for quick switching between the modes. Furthermore, studies using different electrodes for ‘simultaneous’ readouts tend to use one potentiostat equipped with a multiplexer [21,22,23,24], which effectively results in a similar time resolution of each parameter readout as can be achieved by switching modes. Such time resolution is commonly not problematic for biological studies, as the processes in question tend to have longer time scales. On top of that, since most microfabrication methods make use of wafer-scale processes, fabricating one type of electrode generally proves to be much simpler and cheaper than incorporating different kinds of electrodes. Therefore, should truly simultaneous readings for different parameters be necessary, it is still more convenient to fabricate multiple electrodes of one material to sense the different species. Therefore, for non-invasive sensing operations, there are practically no downsides to diversifying the functionality of an electrode. We envision this functionality to be employed (in sequential or simultaneous manner) with other (electrochemical) techniques in many in vitro cardiomyocytes studies (such as the metabolism study), to afford additional information at practically no disadvantage.

EIS is a technique which measures the impedance of an electrochemical cell as a response to an applied perturbative (alternating current) frequency. While it is a perturbative technique, the applied signal is very low in amplitude and therefore cause practically no (redox) change in the system. EIS has been established as a non-invasive, label-free technique in monitoring cells’ mechanical behavior or their physical features [14,25,26,27,28] as well as their (cytoplasmic) chemical features [29,30]. Comparable information on beating/contractile cells can also be provided by analyzing video/visual data [14]. While both techniques can operate with relatively simple instrumentations, electrical data from EIS measurements are much smaller in size, which allows for more straightforward data analysis. For instance, the analysis of the visual data in this work required manual selection of the area of interest, while the electrical data could be directly analyzed as a whole. On top of it all, it offers valuable (extra) information to studies which are already employing similar electrochemical setups. Therefore, in this work, we explore how the RuOx electrode can be operated to monitor the (mechanical) activities of hPSC-CMs.

## 2. Materials and Methods

### 2.1. Setups

The experiments aimed to show a recording of hPSC-CMs activity using our RuOx electrode. However, since the RuOx electrode chip required the use of a non-transparent Teflon chip holder, no visual data could be recorded at the same time to serve as confirmation/validation. Therefore, we used two setups in this experiment: first, an optically-transparent well plate with Pt wire electrodes, in which we recorded both electrical and visual data. Then, the electrical measurement was repeated in a Teflon chip holder with the RuOx electrode.

#### 2.1.1. Well Plate

A coiled (~2 cm) Pt wire (Alfa Aesar, Ø0.25 mm) was fixed onto the bottom of a well in a 12-well plate (Corning, clear, untreated) using a drop of medical-grade epoxy (Loctite hysol M-31CL). The epoxy was then cured in 60 °C oven for at least one hour. Similar coiled Pt wire was also fixed on the lid of the well plate using a piece of tape. It was done in such a way so that the Pt wire would be suspended above the Transwell insert membrane at ~5 mm distance. The prepared setup was rinsed with 70% ethanol three times and kept in a laminar flow hood until measurement.

#### 2.1.2. Teflon Chip Holder

**Figure 1:**
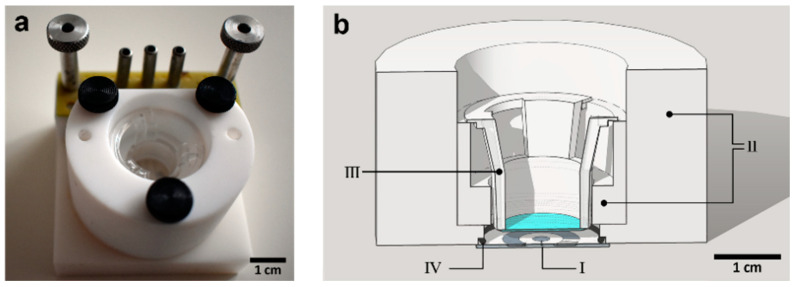
(**a**) Photograph of the chip- and Transwell insert-holder used in the experiment using RuOx electrode. Chip connection block equipped with pogo pins is used to connect the on-chip RuOx electrode to the impedance spectroscope. (**b**) Illustration of the cross-sectional view of the setup. Chip- and Transwell insert-holder (II) suspends the Transwell insert’s (III) membrane above the glass chip (I), with O-ring (IV) to seal the electrochemical cell. Cells were cultured on the top side of the membrane.

A sputtered Pt electrode (circular, Ø2.4 mm) on a glass-chip with ruthenium oxide (RuOx) nanorods modification was used as one of the electrodes in this experiment. The RuOx modification was done by dropping ~5 μL Ru(OH)_3_ suspension in DI water and subsequently heating the chip at 350 °C for 4 h. The chip was used in a Teflon chip holder (Figure 1a) where the hPSC-CMs were cultured on the membrane (colored blue in Figure 1b) and therefore could be suspended on top of the RuOx electrode at ~2 mm distance. A Pt wire (Alfa Aesar, Ø0.25 mm) was used as the other electrode, suspended in the apical side of the Transwell insert’s membrane, at ~5 mm distance. The setup (along with the Pt wire) was autoclaved and kept in a laminar flow hood until measurement. The RuOx electrode on the glass chip was connected with a connection block fitted with pogo pins.

#### 2.1.3. RuOx Electrode

**Figure 2:**
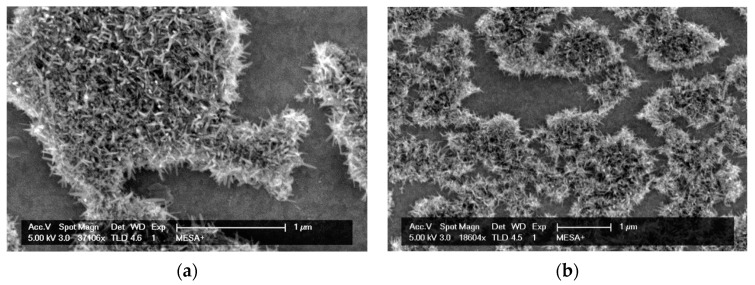
(**a**,**b**) SEM images of typical resulting RuOx nanorods. The nanorods grew on the precursor islands, with typical width between 15–25 nm and length between 110–150 nm.

The RuOx modification was applied on sputtered Pt electrode (Ø2.4 mm, ~200 nm thick) on a glass chip. The fabrication followed the same protocol previous reported [9,11], modified from an original protocol published by Chen, et al. [31] In short, Ru(OH)_3_ precursor was precipitated from 5 mM RuCl_3_ (Aldrich) solution, by adding 5 mM NaOH (Aldrich) solution drop by drop. The resulting precipitation were resuspended in ultrapure water and was dropped onto clean Pt electrode. Once dried, the chip was heated in a pre-heated oven at 350 °C for 4 h under atmospheric air and left to cool down to room temperature overnight. The resulting RuOx nanorods were imaged by scanning electron microscopy (SEM) using FEI Sirion HR-SEM. The amorphous Ru(OH)_3_ precursor formed island-like structures. After the annealing process, RuO_2_ crystalline grew to form rods of 15–25 nm wide and 110–150 nm long on the island-like structures as shown in Figure 2a,b.

### 2.2. Cells Seeding and Culture

This work used hPSC-CMs which were derived from the double-reporter mRubyII_α-Actinin/green fluorescence protein_NKX2.5 (DRRAGN) cell line [32,33]. Transwell inserts (Corning Transwell, 12 mm diameter, polyester membrane with 0.4 μm pores) were coated with Matrigel at 8.3 μg/cm^2^ density (Corning) before seeding. The cells were seeded on the top side of a Transwell insert membrane at 535,000 cells/cm^2^ density. The seeded inserts were cultured until the measurement in an incubator (5% CO_2_, 37 °C) with 500 μL CM-TDI maturation medium [33], refreshed every 48 h. Right before measurement, a cell monolayer was taken out of the incubator and put into the setup with 700 μL CM-TDI medium in it. It was also made sure that no air bubbles were present between the electrode and the membrane. Both the Pt and RuOx materials have been previously reported to be biocompatible in in vitro settings [9,12,25,34]. Therefore, cells viability was observed based solely on their contractile activities.

### 2.3. Impedance and Visual Recording

Setup characterization was done using Bio-Logic SP300 bipotentiostat. The measurements with hPSC-CMs were done using Zurich Instruments’ HF2IS impedance spectroscope in combination with a HF2TA pre-amplifier, controlled via ziControl user interface. 0.1 V peak-to-peak input signal was applied at 100 kHz. The visual data were recorded using a Leica DM IRM inverted microscope equipped with a Leica DFC 300 FX high-speed camera. The video was recorded at 100 frames/s. All measurements were done in room temperature (21 ± 1 °C). The measurements were done in fresh CM-TDI medium to ensure repeatability. Fresh CM-TDI medium had conductivity of 9.25 mS/cm (at 21.5 °C) as measured using Mettler-Toledo LE703 conductivity probe on SevenMulti unit. The fitting of equivalent circuits were performed using ZView version 3.5 h. Other data analyses were done using MATLAB (R2016b).

## 3. Results and Discussion

### 3.1. Measurement Frequency

**Figure 3:**
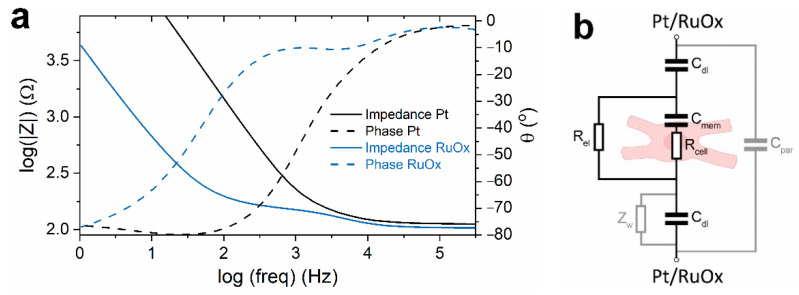
(**a**) Frequency dependence of the system’s impedance in the presence of empty Transwell insert, measured in the hPSC-CMs medium at room temperature. (**b**) Expected equivalent circuit of the system with the hPSC-CMs in place and the (open) Warburg element as surface porosity model in the case of the RuOx nanorods. Abbreviations: C_dl_, double layer capacitance; R_el_, electrolyte resistance; C_mem_, cell membrane capacitance; R_cell_, cytoplasm resistance; C_par_, parasitic capacitance; Z_w_, Warburg element.

In determining the appropriate signal frequency at which we can measure the beating of cardiomyocytes, the setups were first characterized. Figure 3a shows the frequency response of the setups recorded in the presence of an empty Transwell insert in the hPSC-CMs medium at room temperature. The frequency response of the setup using Pt wires resembled a typical two-electrodes system in a liquid-filled chamber/channel [26,35]. At lower frequencies (up to ~100 Hz), the system showed a capacitive behavior, showing the influence of the double-layer capacitance of the electrode-electrolyte interfaces. At higher frequencies (10–300 kHz), the system showed resistive behavior, which corresponded to the electrolyte resistance between the electrodes. The frequency response of the setup using RuOx electrode and a Pt wire showed similar behavior; however, with seemingly an additional relaxation mechanism at the intermediate frequencies (between 5–1000 Hz). An impedance sweep of a flat RuOx electrode [12] did not show similar additional relaxation mechanism. This implies that this extra mechanism corresponds to the rough surface of our RuOx electrode. As shown in Supplementary Materials, the addition of a Warburg element for a porous electrode can model the measured frequency response quite well.

Based on existing literatures [26,28,29], the equivalent circuit of the system (once the cells were introduced) can be predicted (shown in Figure 3b). During contraction, the cardiomyocytes displace their membrane and organelles along with their surroundings [14]. Therefore, the shape change (relative to their relaxed state) is expected to change the volume fraction between the electrodes. Cell membranes have been reported to behave as an insulator at frequencies below 1 MHz, allowing cells to be modelled as insulating spheres at those frequencies [26,35]. Therefore, as the cardiomyocytes contract and change in shape (e.g., thickness), some conductive electrolyte (R_el_) is displaced from the electric field and temporarily replaced by more of the insulating sphere. This event should then be reflected by a change in the measured resistance between the electrodes. For this reason, it is desired to perform the EIS measurement at frequencies between 10–300 kHz. Impedance measured at these frequencies correspond to the resistance between the electrodes, and cell membrane behaves as an insulator.

Moreover, the beating is also expected to change the membrane’s physical properties and, therefore, its capacitance. A study [35] has shown that the change in membrane capacitance also has an effect on the measured impedance at frequencies between 100 kHz and 10 MHz. Higher membrane capacitance was simulated to result in a decrease of impedance. How the membrane capacitance of the beating hPSC-CMs’ changes has not yet been reported in detail. It might be the case that an increase in membrane capacitance would unfavorably obscure the higher impedance resulting from the increase in cell size. For this reason, a measurement frequency of 100 kHz was chosen. It is a frequency at which the effect of change in membrane capacitance is relatively low compared to the effect of change in effective cell volume between the electrodes.

### 3.2. Measurement in a Well Plate with Pt Wires

#### 3.2.1. Visual Data

**Figure 4:**
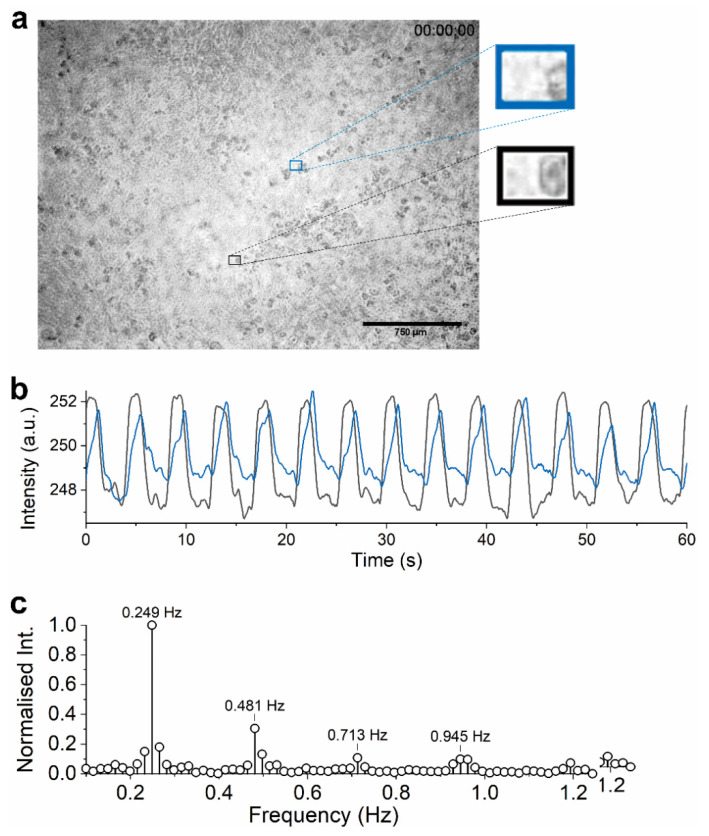
Visual data and their analyses recorded in the well-plate setup. (**a**) Still image of the recorded video of beating hPSC-CMs. Frames note example areas (35 by 25 pixels; corresponding to 85 by 60 μm) that were analysed. (**b**) Mean intensity over time of the two areas noted in (**a**), filtered by moving average (N = 50 frames, corresponding to 0.5 s integration time) for clarity. The black frame on the image corresponds to the black line in this graph, blue frame to the blue line. The graphs have different waveforms as well as a slight phase difference. (**c**) Frequency components of the recorded average intensity of the area in black frame in (**a**) (corresponds to the black graph in (**b**)).

As mentioned, visual data were recorded during measurement in a well plate. They served as a means to validate the electrical data. A still frame of the recorded video is shown in Figure 4a. Five different areas within the video were manually selected and the mean intensity of each area was calculated. The change of the average intensity of each area was then plotted over time.

Examples of the change in intensity of such area are plotted against time in Figure 4b. The resulting graphs show some apparent periodicity. Excluding DC offset, Fourier analysis of the extracted intensity data showed four to five key frequency components between 0.1 and 1.2 Hz. The most prominent frequency component (at 0.249 Hz) was in good agreement with the frequency hPSC-CMs beating was observed visually (observed once every ~4 s or 15 bpm). Such low beat rate was expected for the measurement took place in an uncontrolled environment, outside the incubator. It has been reported that stem cell-derived cardiomyocytes can decrease their contraction rate well below 20 bpm in room temperature and under atmospheric gas [14].

The higher harmonics in Figure 4c seem to make for the signal shape, as the time-domain signal did not appear in a sinusoidal waveform. Fourier analysis of other areas showed some differences in the higher harmonics (also varied waveforms, depending on how the area was chosen); however, the frequency component at 0.249 Hz appeared in all sampled areas. All in all, frequency analysis proved to offer a reliable beating frequency information of the hPSC-CMs monolayer.

Spontaneously beating cultured cardiomyocytes (hPSC-CMs included) have been reported to synchronize their beating frequency and create a stable beating frequency across the monolayer [36,37,38]. It is consistent with the behavior observed of the hPSC-CMs monolayer in our study, comparable beating frequency was observed everywhere in the frame. However, the beating seemed to propagate throughout the frame. Therefore, different places in the frame have a slight phase variance. Figure 4b plots the mean intensity of two different areas (~1 mm apart) on the captured frame over time. One of the areas was found to beat as much as one second earlier than the other. As it was not possible (with the current setup) to localize the visual and electrical observation to the same site, it is not possible to relate any specific part of the visualized beating cycle to its electrical data counterpart.

#### 3.2.2. Electrical Data

**Figure 5:**
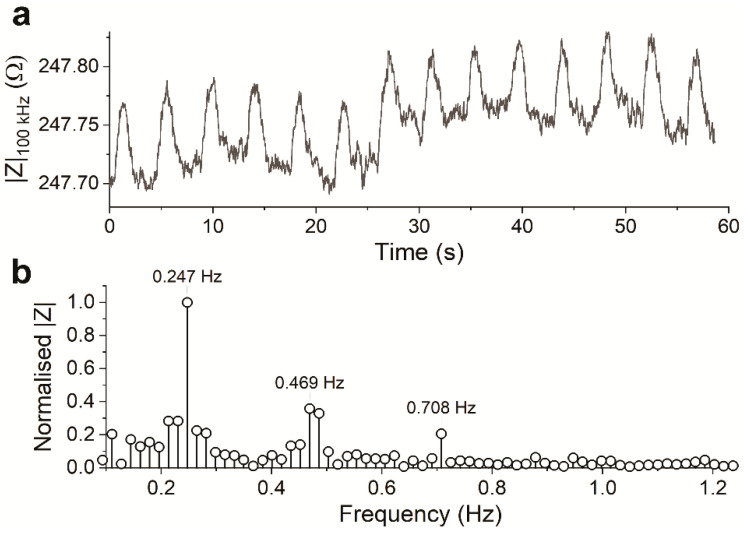
(**a**) The magnitude of the recorded impedance of the contractile hPSC-CMs, recorded by Pt wires in the well-plate setup, plotted over time. (**b**) Frequency components of the recorded magnitude data shown in (**a**).

The electrical data below were recorded at the same time as the visual data presented in the previous section. The impedance between the electrodes was monitored continuously while the hPSC-CMs were contracting and relaxing within the electric field, similar to other reports [25,26]. The recorded magnitude in the presence of beating hPSC-CMs (plotted over time in Figure 5a) was in the expected range based on the measured CM-TDI medium conductivity, as well as the information obtained from setup characterization. Similar to the visual data (Figure 4b), the signal showed apparent periodicity when plotted over time. Fourier-analysis of the recorded magnitude signal (Figure 5b) showed considerably similar frequency components to that of the visual data. The difference in sampling frequencies can account for the slight frequency shifts of the main components. The visual data were sampled at 100 Hz, and the electrical data at 3.6 kHz. The different sampling rates resulted in different resolutions of the frequency spectra, and therefore the slight difference in the frequencies of the components. Keeping that in mind, the high similarity of the frequency components between the electrical and visual data can confirm that the two methods observed the similar events of the beating of (an area of) hPSC-CMs.

**Figure 6:**
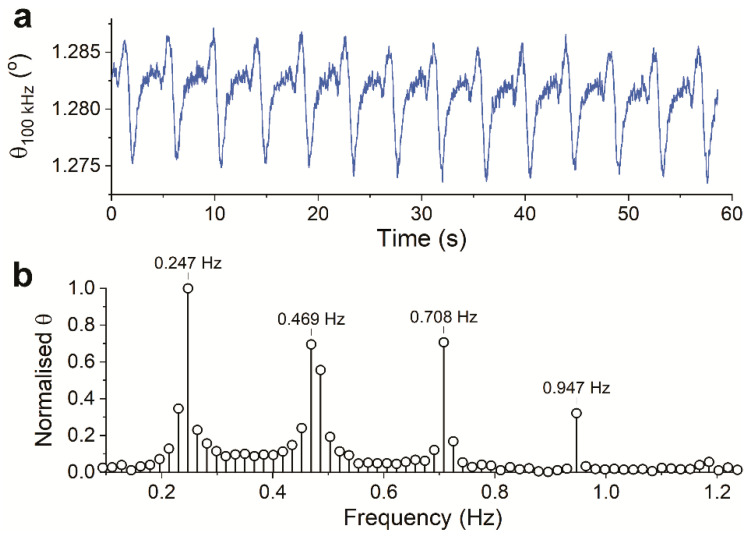
(**a**) The phase of the recorded impedance of the contractile hPSC-CMs, recorded by Pt wires in the well-plate setup, plotted over time. (**b**) Frequency components of the recorded phase data shown in (**a**).

Figure 6a plots the phase of the impedance recorded in the presence of the beating hPSC-CMs. It can be seen that the phase value itself stayed close to zero, which implies that the system at the perturbation frequency was predominantly resistive, as expected from Figure 3a. Nonetheless, the calculated phase still showed small periodic changes at the same frequency (Figure 6b) to that of the calculated magnitude (Figure 5b). A new dynamic (frequency component at 0.955 Hz) was introduced in the phase data. This is reflected in the signal’s different shape, with the notable sharp peak at the end of every periodic signal. Despite the different waveform, from the high similarity and periodicity of the phase data to the magnitude data, it can be concluded that the phase data also observed the same events of beating hPSC-CMs.

Generally, the phase change can be expected to correspond to change in membrane capacitance. Scrutinizing the more complicated waveform of the phase data, it seems that the phase data carried more information than just the change in cell size or displacement (as captured by the visual as well as the magnitude data). Unfortunately, how the recorded phase data correspond to the physical change in beating hPSC-CMs cannot be exacted (based on the gathered data or other studies). Neither can it be predicted how the change in membrane capacitance affected the recorded magnitude signal.

Throughout the measurement, the beating frequency of the hPSC-CMs gradually decreased as the cells’ temperature dropped (from 37 °C in the incubator) to room temperature. As the hPSC-CMs beating slowed down, the observed frequency components also gradually shifted lower. It was captured in the analyses of the prolonged well-plate measurements (Supplementary Materials). Without binning, the frequency components showed broad distributions, which was consistent with the expected frequency shift over time. When the data were split into one-minute bins, the gradual decrease of the frequency components can also be seen, accompanied by a change in signal shape (Supplementary Materials).

The consistency between the visual and electrical data proved 100 kHz to be an appropriate frequency to capture the beating activities of hPSC-CMs, using either the magnitude or phase data.

### 3.3. Measurement in Chip Holder with RuOx Electrode

**Figure 7:**
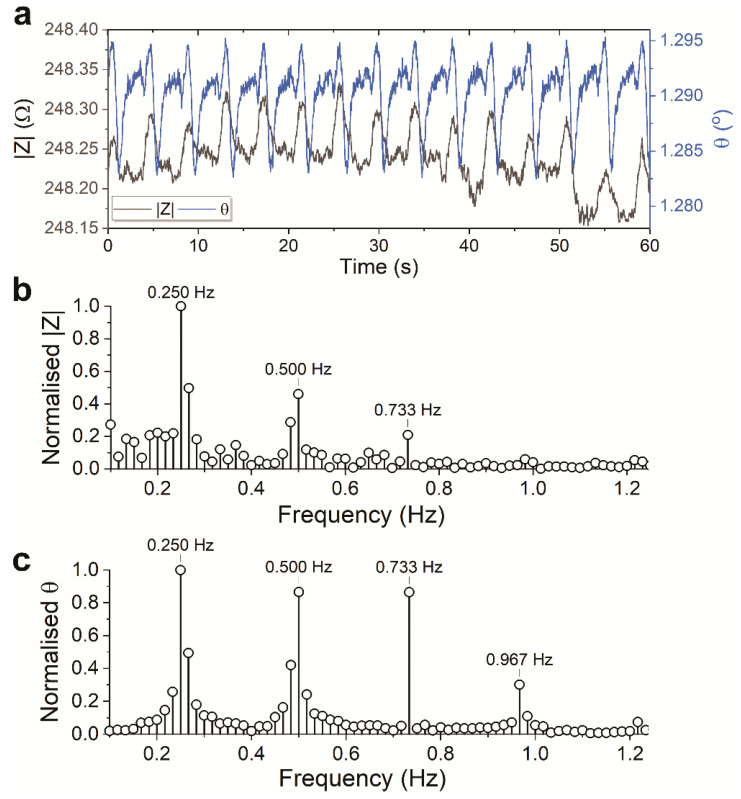
(**a**) Overlaid impedance magnitude and phase of beating hPSC-CMs recorded between RuOx and Pt wire electrodes in the Teflon chip holder setup. (**b**) Frequency components of the magnitude and (**c**) the phase data.

The same measurements were then done using the RuOx electrode in the Teflon chip holder. Figure 7a shows the magnitude and phase data calculated from the impedance recorded using the RuOx electrode.

The waveforms of the signals show remarkable similarity to that recorded using Pt wires. Similarly, Fourier analyses were performed on the gathered data. Figure 7b,c show similar frequency components compared to those presented in Figure 5b and Figure 6b, with a slight frequency difference of the main components. Given the temperature was not controlled during the measurement, a slight temperature difference can explain the minute frequency differences. All in all, the high degree of similarity observed (despite the temperature variance) can conclude that the different setups, as well as the different electrodes, performed comparably.

Compared to the highly localized visual readout, the electrical signal offers information regarding the averaged change of an area. While it did not seem to affect the clarity of the recorded signal, it proved to be problematic for less homogenous cell layer. EIS measurements of clumpy/clustered hPSC-CMs did not deliver any periodic signal; presumably due to the negligible impedance change caused by the cells when compared to the highly conductive ‘holes’ or cell-free paths. However, the RuOx electrodes can be miniaturized to offer improved spatial resolutions, which would allow monitoring of a cell cluster. Simultaneous measurement of multiple electrodes would also offer other relevant information, such as contraction propagation speed and direction.

Furthermore, it has been previously pointed out [14] that cardiomyocytes’ contractility is highly sensitive to its environment. Therefore, measurements in a highly controlled or standardized environment (e.g., inside the incubator) are often indispensable. On-chip sensors offer significant advantage over visual methods for such studies, especially for high-throughput applications. Parallelizing EIS measurements require less cumbersome setups than visual methods. In addition, the resulting electrical signals are also much more straightforward to analyze in parallelized studies than the visual signals. Unlike the electrical signals, there are still many discussions regarding the different methods or standards in visual analyses of cardiomyocytes’ contractility [14,16].

All in all, we have shown that the RuOx electrode can be readily used to monitor the contractile activity of hPSC-CMs monolayers, just as well as conventional Pt wire electrodes and visual analysis. Using simple method, the electrode delivered highly valuable and straightforward information that can be a strategic addition readout for many in vitro hPSC-CMs studies.

## 4. Concluding Remarks

In this work, we have demonstrated and discussed a novel use of the on-chip RuOx electrode in monitoring the contractile activities of hPSC-CMs. The gathered data and their analyses confirmed that EIS measurement at 100 kHz could capture the contractile events of the hPSC-CMs, both from the magnitude as well as the phase data. The magnitude data accurately resembled the visual data both in the waveform as well as in the frequency components, which most probably is a direct reflection of the changes in cell size. The phase data, on the other hand, could contain information beyond mechanical changes of the biological cells.

The use of Pt wire as the other electrode was chosen to achieve an electric field that surely transversed the hPSC-CMs monolayer, similar to that achieved in the well plate setup. However, using another electrode on the chip (the Pt counter electrode or the Ag/AgCl reference electrode) would simplify the setup. Therefore, it might be a worthy undertaking in simulating and testing whether the resulting electric field between two on-chip electrodes in the planar configuration would transverse the cell culture plane.

This mode of operation adds to the versatility of the RuOx electrode’s use in in vitro studies. The added functionality of RuOx in monitoring the contractile hPSC-CMs can provide information on the cells’ activity while studying other parameters.

## Data Availability

Data sharing is not applicable for this article.

## Supplementary Materials

The following are available online at https://www.mdpi.com/1424-8220/21/4/1433/s1, Figures S1 and S2: Fitting of EIS data with equivalent circuit; Figures S3–S5: Frequency shifts over time; Figure S6: Larger image of the still image of video recording, Video: Recording of the contractile hPSC-CMs.
