# Supplementary material for: Monitoring Contractile Cardiomyocytes via Impedance Using Multipurpose Thin Film Ruthenium Oxide Electrodes
Source: Sensors (Basel). 2021 Feb 18;21(4):1433. doi: 10.3390/s21041433 (PMC7923073; doi:10.3390/s21041433)
Supplement: Supplementary file 1 [file sensors-21-01433-s001.zip › sensors-1097557-supplementary.pdf]

Communication

Monitoring Contractile Cardiomyocytes via Impedance Using Multipurpose Thin Film Ruthenium Oxide Electrodes

Esther Tanumihardja [1,*], Douwe S. de Bruijn [1], Rolf H. Slaats [2], Wouter Olthuis [1] and Albert van den Berg [1]

[1] BIOS Lab on a Chip group, Max Planck Centre for Complex Fluid Dynamics and Technical Medical Centre, MESA+ Institute for Nanotechnology, University of Twente, 7500 AE Enschede, The Netherlands; d.s.debruijn@utwente.nl (D.S.d.B.); w.olthuis@utwente.nl (W.O.); a.vandenberg@utwente.nl (A.v.d.B.)

[2] Applied Stem Cell Technologies Group, Technical Medical Centre, University of Twente, 7500 AE Enschede, The Netherlands; r.h.slaats@utwente.nl

* Correspondence: e.tanumihardja@utwente.nl

S1. Fitting of EIS data with equivalent circuit

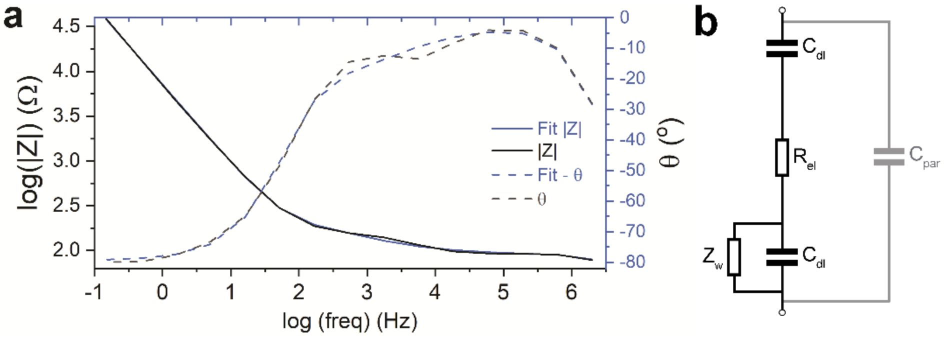

Figure S1. (**a**) Fitting of the measured RuOx impedance in the CM-TDI medium using (**b**) the equivalent circuit model. The resulting fitting parameters values were: Cdl, 76 nF; Rel, 100 Ω; diffusion impedance (Wo-R), 223 Ω; diffusion time (Wo-T), 3 ms; and Wo-P fixed to 0.5. The sum of squared errors between fit and data was 0.028.

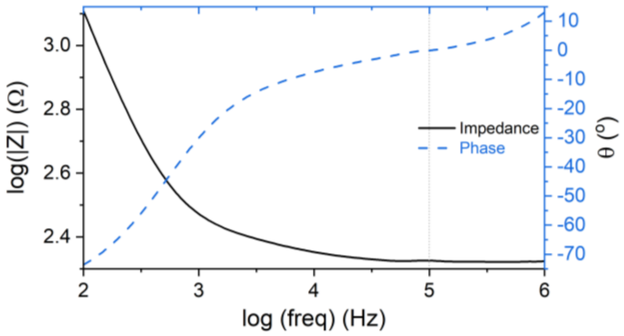

Figure S2. Sweep on the well plate with cells with Pt wires shows similar frequency response. At 100,000 Hz, the response is mostly resistive.

S2. Frequency shifts over time

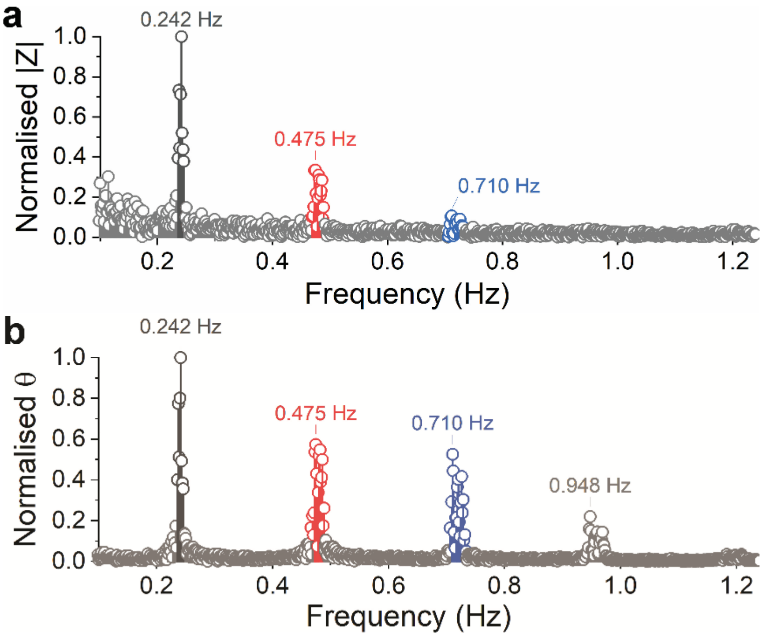

Figure S3. Frequency analyses of the EIS (**a**) magnitude data and (**b**) phase data of the entire 20-minutes recording on the well-plate setup. Similar frequency components can be seen; however, they show wider distribution due to the change in temperature during the measurement. The coloured frequencies correspond to the frequencies analysed in Figure S6a and Figure S7a.

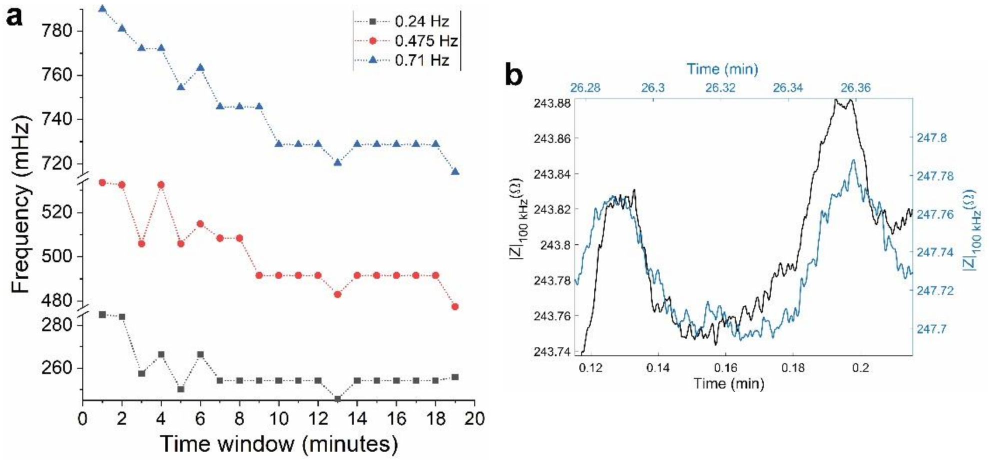

Figure S4. (a) The magnitude data shown in Figure S5, split into 19 one-minute bins Frequency analyses of each bin (plotted over time) show a shift of the frequency components toward lower frequencies. **(b)** EIS magnitude signal at the beginning of the measurement overlaid with the zsignal recorded at the end of the measurement. As the frequency components did not change uniformly, a slight change in the time-domain signal over time can be seen.

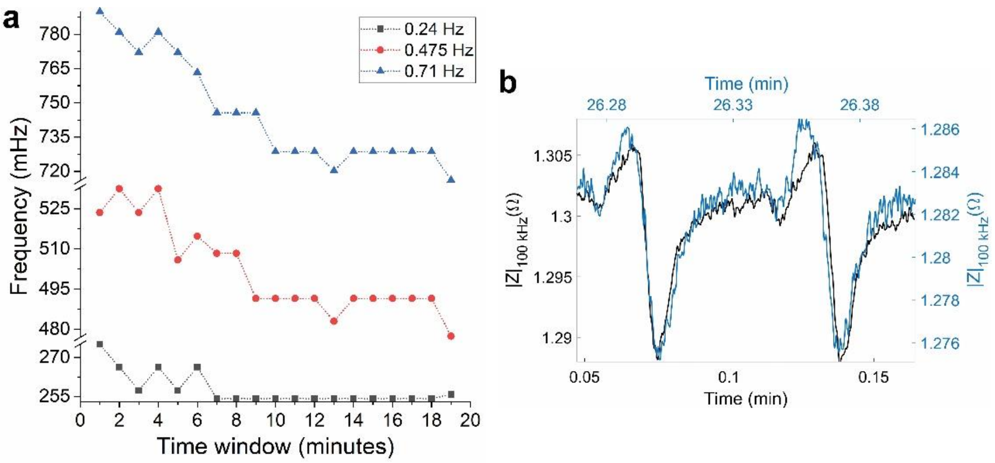

Figure S5. **(a)** The phase data shown in Figure S5, split into 19 one-minute bins. Frequency analyses of each bin (plotted over time) show a shift of the frequency components toward lower frequencies. **(b)** EIS phase signal at the beginning of the measurement overlaid with the signal recorded at the end of the measurement. As the frequency components did not change uniformly, a slight change in the time-domain signal over time can be seen.

S3. Still image of the video recording

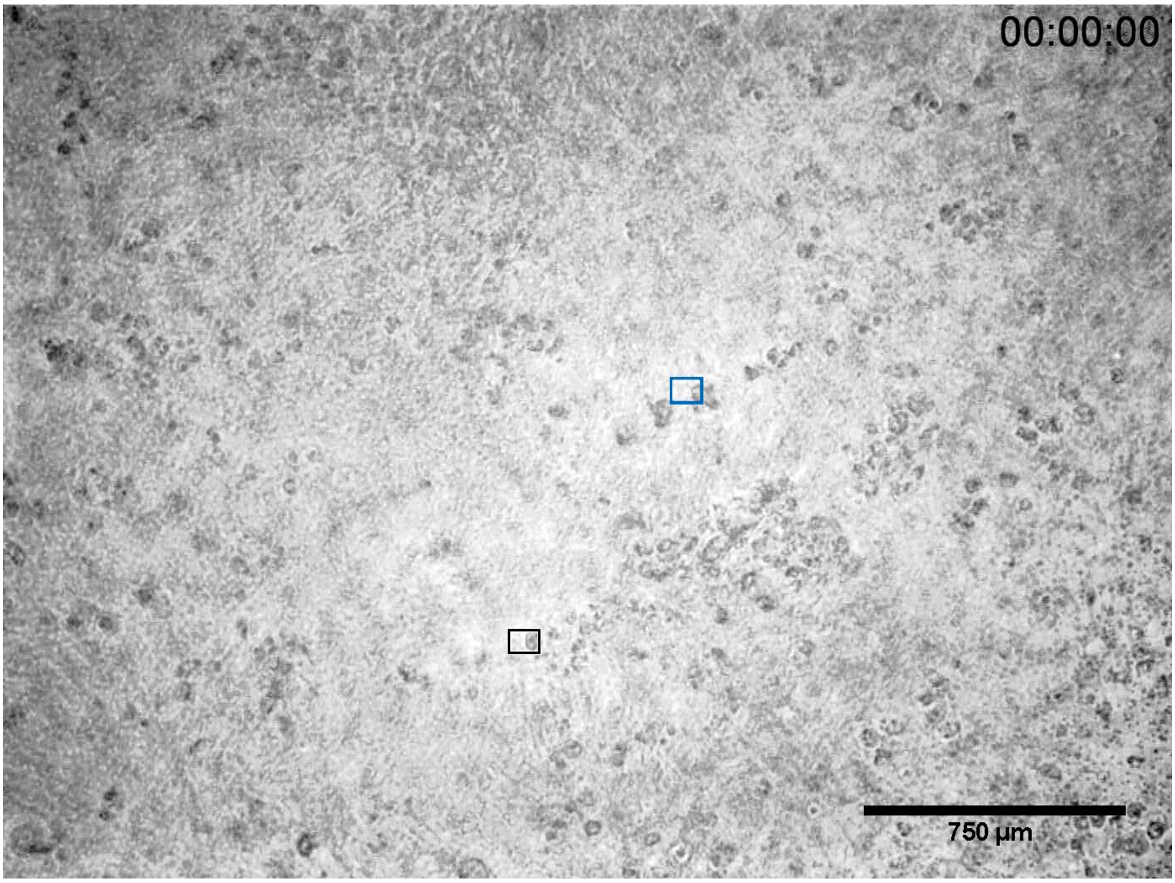

Figure S6. Still image of the recorded video of beating hPSC-CMs. Frames note example areas (35 by 25 pixels; corresponding to 85 by 60 µm) that were analysed.
